# Feasibility of a remote-care-system-based sibling peer event for young carers: a longitudinal pilot study

**DOI:** 10.3389/fpsyg.2026.1940533

**Published:** 2026-09-30

**Authors:** Rie Wakimizu, Keita Sasaki, Mariko Hoshi, Rika Ogoshi, Marise Kasai, Yoshiyuki Kawano

**Affiliations:** 1 Division of Health Innovation and Nursing, Department of Child Health Care Nursing, Institute of Medicine, University of Tsukuba, Tsukuba, Japan; 2 Division of Pediatric Nursing, Faculty of Health Sciences, Hokkaido University, Sapporo, Japan; 3 Graduate School of Comprehensive Human Sciences, University of Tsukuba, Tsukuba, Japan; 4 Institute of Human Sciences, University of Tsukuba, Tsukuba, Japan

**Keywords:** family empowerment, quality of life, remote care system, siblings of children with disabilities, young carers

## Abstract

Siblings of children with chronic conditions or disabilities (young carers) often take on caregiving roles yet remain largely outside family-support frameworks, and intervention research targeting them is scarce, particularly in Japan. This prospective longitudinal pilot study examined the feasibility of delivering an in-person sibling peer event (a one-day group gathering combining recreational activities, peer interaction, activities facilitating emotional expression, and crafts) embedded within a continuously operating online remote care system (RCS) and explored preliminary outcome patterns, rather than testing efficacy. Twenty children participated; because one sibling pair shared a single proxy report, they yielded 19 analysis cases (hereafter, 19 cases), assessed before the event (T0), immediately after (T1), and 3 (T2) and 6 (T3) months later, within a feasibility framework and using the Family Empowerment Scale, the EQ-5D (utility and the 0–100 visual analog scale [VAS]), the short Japanese Zarit Burden Interview (J-ZBI_8), and a social resource utilization item. Recruitment was open with no eligibility restrictions; 20 of 22 applicants attended (participation 91%), and no adverse events were reported at any assessment; acceptability was high, with all children rating the event enjoyable and 94.7% wishing to attend again. Questionnaire data were available for 16, 18, 10, and 12 of 19 cases at T0–T3, illustrating the difficulty of longitudinal follow-up. Outcomes were respondent-level: parents provided proxy reports (10 cases) and siblings self-reports (9 cases), so scores index different constructs and are summarized accordingly rather than as a single pooled measure. Changes in quality of life were small, non-significant, and concentrated among parent-proxy reports; because two non-comparable EQ-5D forms were used, utilities are reported descriptively only. As an exploratory signal warranting monitoring rather than a safety concern, self- and proxy-reported burden scores rose immediately after the event and returned toward baseline by 6 months. This pilot provides preliminary feasibility signals for embedding an in-person sibling event within continuous online support and indicates the need for controlled studies to examine the efficacy of such in-person events for siblings, while highlighting measurement and retention challenges to address in future trials.

## 1 Introduction

Among families living with a child who has a disability or chronic illness (hereafter, a child with a disability), siblings of the child with a disability (hereafter, siblings) have often been left out of family-support frameworks as an “invisible presence.” From early childhood, siblings share daily life with their brother or sister with a disability and take on direct care and supervision, yet they have few opportunities to express their own emotions and difficulties. Prior research in Japan and abroad has shown that siblings are prone to social isolation, psychological burden, and reduced self-efficacy (Stoneman, 2005). As family members who take on caregiving and emotional-support roles for a brother or sister, siblings can also be understood as young carers, a group reported to be at elevated risk of anxiety and depression (Levante et al., 2025). Despite their high support needs, empirical intervention studies targeting siblings remain extremely scarce, particularly in Japan. In Japan, family members often play substantial roles in supporting children with illness or disabilities, and some siblings may also take on caregiving or supportive roles within the family. The present study included siblings of children with a range of chronic conditions and disabilities, including autism spectrum disorder/pervasive developmental disorder, cerebral palsy, chromosomal abnormalities/rare diseases, and intellectual disability/developmental delay (Table 1).

**Table 1:** Participant characteristics.

Characteristic	Value
Sibling’s school level (*N* = 20 children)	Elementary school (grades 1–6), 15; junior/senior high school, 5
Sibling sex (*N* = 20 children)	9 boys, 11 girls
Care recipient’s condition (*N* = 19)	Autism spectrum disorder/pervasive developmental disorder, 7; cerebral palsy, 4; chromosomal abnormality/rare disease, 4; intellectual disability/developmental delay, 3; other, 1
Assessment method (*N* = 19 records)	Parent proxy report, 10; sibling self-report, 9

One case comprised a sibling pair (a first- and a third-grader, both girls) for whom the parent provided a single proxy report applicable to both; thus 20 children contributed 19 outcome records. In the Japanese school system, elementary school corresponds to approximately 6–12 years of age and junior/senior high school to approximately 12–18 years. Assessment method denotes who completed the questionnaire—a parent (proxy report) for younger elementary-school children, or the sibling (self-report) for sixth-grade and junior/senior high-school students. Two cases registered after the event and therefore lack baseline (T0) data.

In recent years, remote care systems (RCS) that use information and communication technology have begun to be introduced to support family caregivers. An RCS provides diverse services online—such as a family empowerment program, peer salons, individual consultations, and webinars—enabling support that transcends geographic and temporal constraints (the RCS used in this study is described in detail in Methods, Section 2.2). The RCS developed and operated by our research group primarily serves siblings of children with disabilities and their parents, and registrants also gain opportunities to participate in in-person sibling events.

Reviews of psychosocial sibling-support programs have reported improvements in self-esteem, social well-being, knowledge of the condition, and emotional and behavioral adjustment following participation (Wolff et al., 2023). Although in-person peer events are expected to provide siblings in similar circumstances with opportunities for interaction and emotional expression, sibling-support programs reported to date have been predominantly in-person, group-based, and episodic, with few embedded within a continuous support infrastructure and only a minority incorporating remote or online components (Al-Hakeem et al., 2025). Episodic in-person contact and continuous, geographically unconstrained support have thus rarely been combined within a single model, and the longitudinal course of such a combined model for siblings (young carers) of children with disabilities has seldom been examined.

In this study, an in-person sibling peer event was embedded within a continuously operating online RCS, coupling episodic in-person connection with continuous remote support. Using longitudinal data at four time points, the primary aim was to assess feasibility—recruitment, participation, retention, questionnaire completion, and safety. Because the RCS is grounded in a family-empowerment framework and supports the sibling within the family unit, we additionally described, as exploratory secondary aims, changes over time in (a) family empowerment (Family Empowerment Scale [FES]), (b) health-related quality of life (EQ-5D), (c) caregiver burden (Japanese short version of the Zarit Burden Interview [J-ZBI_8]), and (d) social resource utilization among RCS-registered siblings and their parents, and explored the association between RCS service use and changes in caregiver burden. Longitudinal evaluations of sibling peer events delivered as an integral part of a continuous online remote care system remain scarce.

## 2 Methods

### 2.1 Study design

We conducted a prospective longitudinal study examining changes in participants’ psychosocial indicators before and after a sibling event. We assessed participants at four time points: before the event (T0), immediately after the event (T1), 3 months later (T2), and 6 months later (T3).

### 2.2 Study setting: the remote care system

The study was conducted within the Remote Care System (RCS), an online platform developed and operated by our research group to support family caregivers of children with chronic conditions or disabilities. Grounded in a family empowerment framework refined through our group’s prior program of research—one component of which, an online family empowerment program, has been evaluated previously (Wakimizu et al., 2022)—the RCS is organized around four core services delivered online: a Family Empowerment Program (FEP), individual professional consultations, peer support salons, and webinars. These services are provided by an interdisciplinary, multi-professional care team—comprising nurses (including certified nurse specialists), physicians, social workers, educators, and psychologists—who together form a nationwide network of supporters (resource persons) and respond flexibly to each family’s needs. By operating online, the RCS extends continuous support beyond the geographic and temporal constraints of conventional in-person services; registered users may also take part in in-person sibling events such as the one evaluated in the present study.

### 2.3 Intervention

The sibling event was held on a single day in the summer of 2025 at a public convention facility in eastern Japan. It was organized by our research group together with the Remote Care System, with the participation of 13 volunteers from collaborating organizations. The event comprised two sessions: an elementary-school session (10:00–12:30) and a junior/senior high-school session (12:30–15:00). The program consisted of three components: recreation, sibling work (emotional expression using a rope visual analog scale [VAS]), and craft activities. The ordering was intentional: recreation served as an icebreaker to ease emotional expression during the rope-VAS activity, and craft-making was placed last in consideration of the psychological load that can follow emotional expression. The event aimed to provide siblings with opportunities for peer interaction and emotional expression; it was not designed as a clinical intervention targeting a prespecified measurable outcome. The rope-VAS activity was intended to facilitate emotional expression as an intervention component, not as an outcome measure, and emotional responses during the activity were not systematically assessed as study outcomes.

### 2.4 Participants

The unit of analysis was one case per participating sibling. Participants were not randomly sampled; RCS registrants who volunteered for the in-person event were included (self-selected). At the time of data collection, 159 caregivers were registered with the RCS. Twenty sibling children participated in the sibling event; no participant was excluded from the analysis. One case comprised a sibling pair (a first-grader and a third-grader, both girls) for whom the parent completed a single proxy report that, the parent confirmed, applied to both children; the 20 children therefore yielded 19 case records. Of the 20 children, 15 were elementary-school-aged and 5 were junior/senior high-school-aged; among the 19 records, a parent provided the assessment (proxy report) in 10 cases and the sibling did so (self-report) in 9 (Table 1). Because respondents differed, the outcome measures are respondent-level: proxy reports index the parent’s perspective (including, for the J-ZBI_8, the parent’s own perceived burden) and self-reports the sibling’s, so the two are not equivalent and are treated as an exploratory pooled summary rather than a single homogeneous outcome. The number of valid responses to the follow-up questionnaire was 16 cases at T0, 18 at T1, 10 at T2, and 12 at T3. The 15 cases with valid responses at both T0 and T1 constituted the analytic sample for the paired t tests.

### 2.5 Assessment method

In accordance with the RCS terms of use, the sibling completed the assessments directly (self-report) when old enough to do so—sixth-grade elementary and junior/senior high-school students (9 cases)—whereas a parent provided a proxy report for younger elementary-school children (10 cases). Scores for proxy cases reflect the sibling’s status as perceived by the parent. Because the respondent differed across cases, all psychosocial outcomes should be interpreted as perceived QoL and perceived caregiver burden at the respondent level rather than as objective indicators of the sibling’s status.

### 2.6 Measures

#### 2.6.1 Family Empowerment Scale

Family empowerment was assessed with the 10-item short form of the Japanese FES (Sato et al., 2023), which was developed using item response theory from the 34-item Japanese version of the FES (Wakimizu et al., 2010), itself adapted from the original Family Empowerment Scale (Koren et al., 1992). Items are rated on a 5-point scale, yielding a total score from 10 to 50, with higher scores indicating greater family empowerment.

#### 2.6.2 Health-related quality of life (EQ-5D)

The RCS administered an age-appropriate EQ-5D form (EuroQol Group, 1990): adolescents who self-reported under their own (minor) account received the youth version (EQ-5D-Y; three levels), whereas parents completing proxy reports for younger children received the adult version (EQ-5D-5L; five levels). Utility values were therefore calculated with instrument-specific Japanese value sets—EQ-5D-Y responses with the value set of Shiroiwa et al. (2021) and EQ-5D-5L responses with that of Ikeda et al. (2015; constant term = −0.060924). Because the data export relabeled all responses with adult-version wording, form assignment was determined from each respondent’s age at assessment. The two value sets are anchored on different scales and are not directly comparable. The mean EQ-5D-5L value at registration among all RCS registrants in this study (*n* = 159) was 0.791.

#### 2.6.3 Zarit Burden Interview, Japanese short version (J-ZBI_8)

The J-ZBI 8 (8 items, 0-32 points), the Japanese short version (Arai et al., 2003) of the Zarit Burden Interview (Zarit et al., 1980), was used; higher scores indicate greater caregiver burden. Although the J-ZBI was originally developed for caregivers of older adults, it is widely used as a generic measure of subjective caregiving burden; here we used it to capture the perceived burden of the responding caregiver, and its applicability to sibling caregivers is acknowledged as a limitation.

#### 2.6.4 Social resource utilization

A single item rated on a 4-point scale ranging from “not utilized at all” (1) to “well utilized” (4).

#### 2.6.5 RCS service use

For the four services (FEP, peer salon, individual consultation, and webinar), participation (yes/no) and the number of services used (0–4) were coded from system logs as of March 17, 2026; this variable represents cumulative use accrued after the event, as the large majority of participants registered in connection with the event.

### 2.7 Statistical analysis

Consistent with its pilot design, this study primarily aimed to assess the feasibility of delivering an RCS-embedded sibling event—recruitment, participation, retention, questionnaire completion, and safety—rather than to test efficacy; outcome analyses were exploratory and intended to inform future trials. The study is reported in line with the TREND statement for non-randomized evaluations and, where applicable, with the reporting principles of the CONSORT extension for randomized pilot and feasibility trials (Eldridge et al., 2016). We calculated descriptive statistics (means and standard deviations) for each indicator by time point. Follow-up retention was defined as the return of a completed questionnaire at a given time point; returned questionnaires provided usable data on all scales, so the counts reported (16, 18, 10, and 12 of 19) reflect the number of questionnaires returned at each time point, the lower value at T2 reflecting fewer returns rather than any scale-specific missingness. For the EQ-5D, because the youth (EQ-5D-Y) and adult (EQ-5D-5L) forms use value sets anchored on different scales, utility values are summarized descriptively by respondent type and are not pooled or tested inferentially; the EQ-5D VAS (0–100), which is common to both forms, is used as the comparable quality-of-life descriptor. For FES, J-ZBI_8, social resource utilization, and the EQ-5D VAS, we examined exploratory changes from T0 to T1 using paired *t* tests and Cohen’s d (small, d = 0.20; medium, d = 0.50; large, d = 0.80). The J-ZBI_8, although developed primarily for caregivers of older adults, was used in the absence of an established Japanese burden measure for young carers; this limitation is considered in the Discussion. As further exploratory analyses, we (a) compared RCS service use between cases in which J-ZBI_8 increased from T0 to T1 and cases in which it decreased or remained unchanged, using Fisher’s exact test; and (b) classified care recipients by required level of assistance—near-total/total assistance as the severe group and none/partial assistance as the mild group—and compared indicators between groups (Mann–Whitney U test). Because this was an exploratory pilot, no adjustment for multiple comparisons was made and all inferential results are hypothesis-generating. We set the significance level at two-sided 5%. Analyses were conducted in Python (scipy, scikit-learn). Analysis code is available from the corresponding author on reasonable request.

### 2.8 Ethical considerations

We conducted this study in accordance with the 1964 Declaration of Helsinki and its later amendments, with the approval of the Research Ethics Committee of the Institute of Medicine, University of Tsukuba (approval no. 1830-1). Participants (parents and siblings aged 12 years or older) were informed about the study’s purpose, methods, the voluntary nature of participation, and the protection of personal information, and written consent was obtained. For children under 12 years of age, a parent registered and provided consent in accordance with the RCS terms of use; participation in the event was voluntary, and children were free to decline or to stop participating at any time. Data were managed anonymously.

## 3 Results

### 3.1 Feasibility and process indicators

Recruitment was open, with no eligibility restrictions or capacity limit. Of 22 children who applied, 20 attended the event (two were absent on the event day; participation rate 91%), yielding 19 analysis cases (Figure 1). Outcome-questionnaire data were available for 16 of 19 cases at T0 (84%), 18 at T1 (95%), 10 at T2 (53%), and 12 at T3 (63%); the lower count at T2 reflects fewer questionnaires returned at that time point. Despite reminders, questionnaire response declined over follow-up; reasons for non-response were not systematically recorded, so the causes of attrition could not be determined. Of the 20 participants, 2 had used the RCS before the event, whereas the remaining 18 registered in connection with it; 9 of 20 (45%) had used at least one of the four RCS services (FEP, peer salon, individual consultation, or webinar) after the event (cumulative use, as of the March 2026 data extraction). The day-of post-event acceptability survey was completed by all 19 participating children (100%) and by 12 parents. No adverse events were reported by any participant at the post-event, 3-month, or 6-month assessments. All planned program components (recreation activities, the craft/snack-bag activity, and the sibling-sharing activity) were delivered as scheduled, and no participant withdrew during the event.

**Figure 1:**
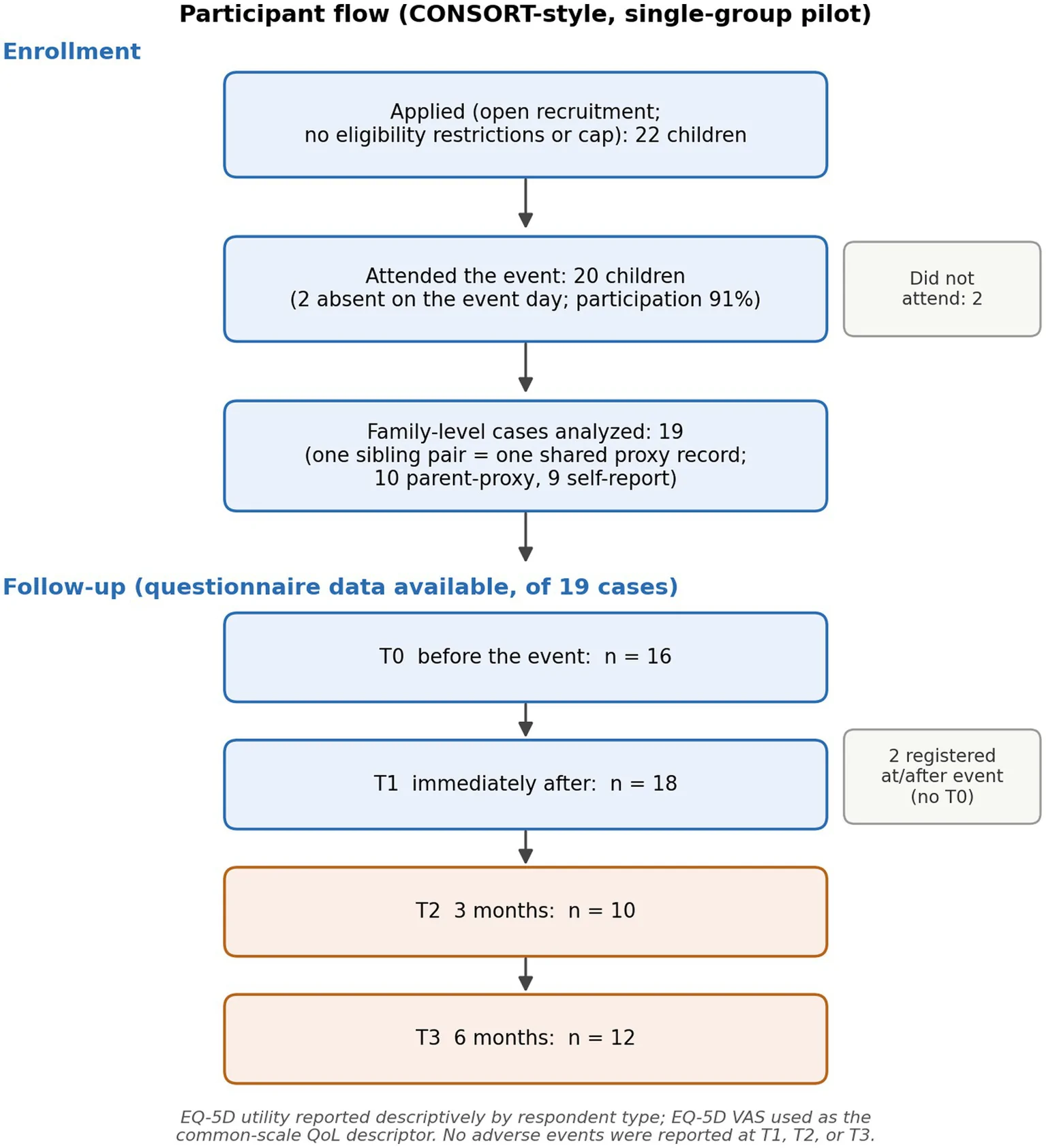
Participant flow (CONSORT-style, single-group pilot). Of 22 children who applied (open recruitment, no eligibility restrictions), 20 attended (two were absent on the event day; participation 91%), yielding 19 family-level cases (one sibling pair = one shared proxy record). Questionnaire data were available for 16, 18, 10, and 12 of 19 cases at T0–T3. Two families registered with the RCS at or after the event (no T0). No adverse events were reported at any assessment.

### 3.2 Acceptability

Among the 19 children, all rated the event as enjoyable (94.7% “very fun,” 5.3% “fun”), and 94.7% wanted to attend again (57.9% “yes,” 36.8% “if possible”), with no negative responses. Among the 12 parents, all rated the venue atmosphere and staff positively (100% “good” or “very good”) and perceived their child as satisfied (100%), and 91.7% were themselves satisfied, with no dissatisfied responses; free-text comments frequently requested continued or regular sibling gatherings.

### 3.3 Participant overview

In total, 20 sibling children participated in the event: 15 elementary-school children (grades 1–6) and 5 junior/senior high-school students. In the Japanese school system, elementary school corresponds to approximately 6–12 years of age and junior/senior high school to approximately 12–18 years. The care recipients’ diagnoses were autism spectrum disorder/pervasive developmental disorder (*n* = 7), cerebral palsy (*n* = 4), chromosomal abnormalities/rare diseases (*n* = 4), intellectual disability/developmental delay (*n* = 3), and other (*n* = 1). Regarding required level of assistance, 10 were severe (near-total/total assistance) and 9 were mild (none/partial assistance)—roughly evenly split—and 4 cases required medical care. The post-event participant survey administered on the day (19 cases) and the parent survey (12 responses) were predominantly positive (Table 2).

**Table 2:** Feasibility and acceptability indicators.

Domain	Indicator	*n*/*N*	Result	Benchmark (*post hoc*)	Assessment
Recruitment	Open enrollment (no eligibility limit or cap)	–	–	–	Feasible
Participation	Attended/applicants	20/22	91%	≥80%	Met
Questionnaire completion	T0 (before event)	16/19	84%	≥70%	Met
	T1 (immediately after)	18/19	95%	≥70%	Met
	T2 (3 months)	10/19	53%	≥70%	Not met
	T3 (6 months)	12/19	63%	≥70%	Not met
Acceptability	Rated event enjoyable (children)	19/19	100%	≥75%	Met
	Wished to attend again (children)	18/19	94.7%	≥75%	Met
Safety	Adverse events	0/20	0%	–	Feasible
Fidelity	Planned components delivered as scheduled	–	Yes	–	Feasible
Exposure	Any RCS service use after event (4 services, cumulative)	9/20	45%	–	Descriptive

Benchmarks are post hoc reference thresholds (not prespecified) drawn from the pilot and feasibility trial literature (Eldridge et al., 2016; Herbert et al., 2019); targets vary across studies, and “Met/Not met” labels are interpretive only. Participation = attended/applicants. Questionnaire completion = return of usable data for the relevant scales at each time point, as a proportion of the 19 analysis cases; the lower value at T2 reflects fewer questionnaires returned at that time point. Acceptability is from the on-site post-event survey (children, *n* = 19). RCS service use = cumulative engagement with any of the four RCS services (FEP, peer salon, individual consultation, or webinar) after the event, as recorded in the March 2026 system log.

### 3.4 Descriptive statistics for each indicator

Descriptive statistics by time point are shown in Table 3. Because respondents differed across cases, tabulated values represent an exploratory pooled summary across parent-proxy and sibling self-reports and should be read as respondent-level rather than as a single homogeneous outcome. The four-time-point change in J-ZBI_8 (T0: 9.0 → T1: 14.1 → T2: 15.3 → T3: 8.2) showed a nonlinear, inverted-U pattern (Figure 2).

**Table 3:** Descriptive statistics for each indicator at each time point (M ± SD).

Indicator	Before event (*n* = 16)	After event (*n* = 18)	3 months (*n* = 10)	6 months (*n* = 12)
FES (10–50)	30.9 ± 10.0	30.6 ± 9.3	31.8 ± 6.7	32.9 ± 9.2
EQ-5D VAS (0–100)	73.1 ± 17.3	78.8 ± 15.3	76.9 ± 14.2	72.8 ± 25.1
J-ZBI_8 (0–32)	9.0 ± 4.7	14.1 ± 8.9	15.3 ± 8.1	8.2 ± 5.6
Social resource utilization (1–4)	2.94 ± 0.85	2.89 ± 0.96	2.90 ± 0.74	3.08 ± 0.67

Valid-response counts were 16, 18, 10, and 12 at T0, T1, T2, and T3, respectively, for all measures, including EQ-5D utility. Values integrate parent proxy reports (10 cases) and sibling self-reports (9 cases). The EQ-5D VAS (0–100), common to both instrument forms, is shown as the comparable quality-of-life descriptor; EQ-5D utility is not tabulated by time point because the youth and adult value sets are not comparable, and respondent-stratified utility changes are reported in the text.

**Figure 2:**
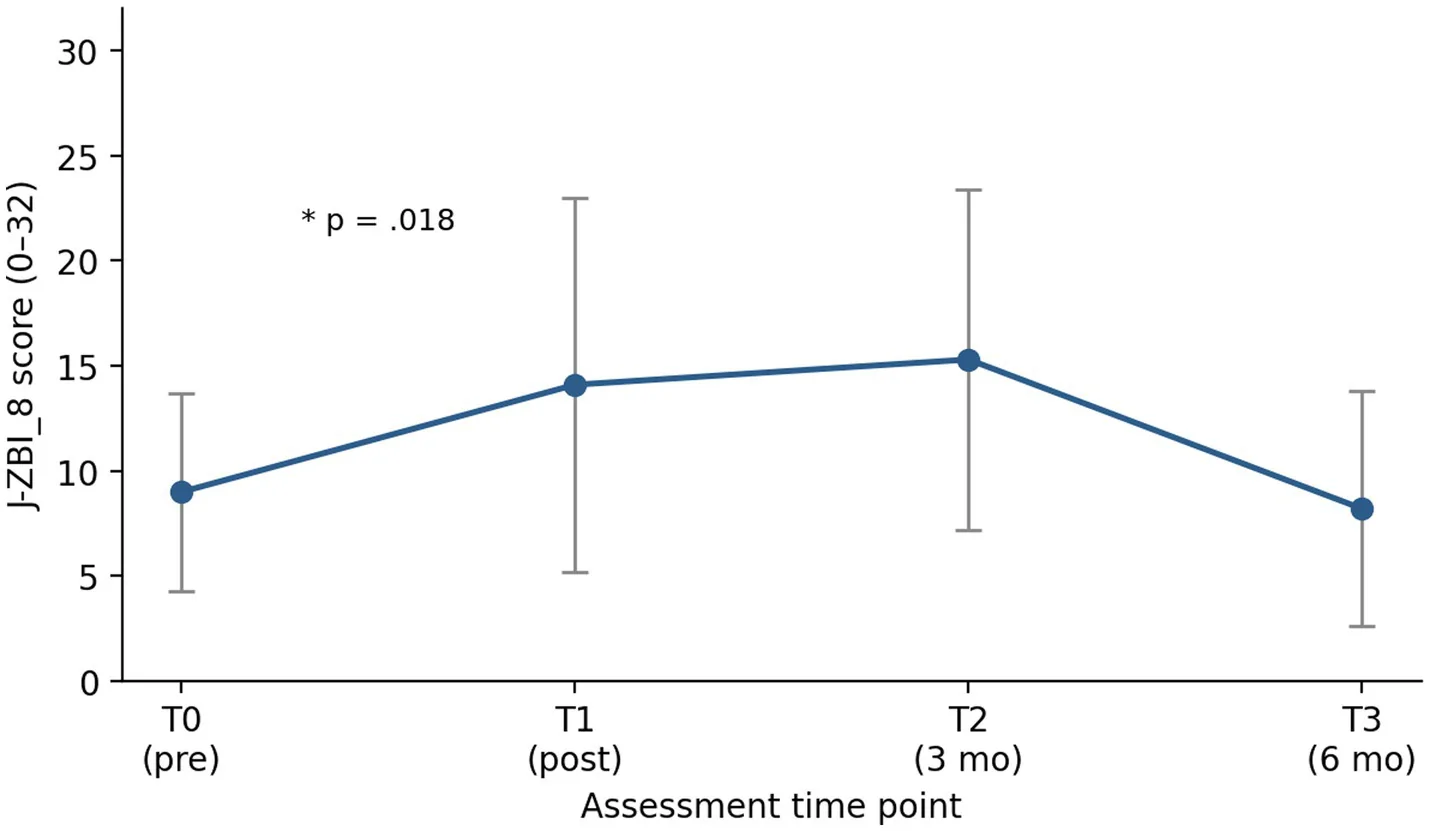
Four-time-point change in J-ZBI_8 Scores (M ± SD). The vertical axis shows the J-ZBI_8 score (0–32) and the horizontal axis the assessment time point. Error bars indicate standard deviations. Because the number of respondents differs across time points (*n* = 16, 18, 10, and 12) and follow-up attrition was non-random, the plotted values are descriptive means from differing subsamples connected for illustration; they should not be read as a within-person longitudinal trajectory. T0: 9.0 ± 4.7 (*n* = 16), T1: 14.1 ± 8.9 (*n* = 18), T2: 15.3 ± 8.1 (*n* = 10), T3: 8.2 ± 5.6 (*n* = 12). The asterisk denotes *p* = 0.018 for the paired T0–T1 comparison (*n* = 15; Table 4).

### 3.5 Exploratory change from T0 to T1

Given the small sample and heterogeneity in respondent type, the following paired comparisons are exploratory and hypothesis-generating; effect sizes and confidence intervals are emphasized over significance testing. Exploratory paired comparisons (*n* = 15 pairs) are shown in Table 4. The EQ-5D VAS showed a non-significant change from T0 (72.3 ± 17.6) to T1 (80.3 ± 14.6) (Δ = +7.9, *t*(14) = +1.13, *p* = 0.28, d = 0.29). J-ZBI_8 increased immediately after the event (Δ = +4.73, *t*(14) = +2.69, *p* = 0.018, d = 0.69). No significant changes were found for FES (Δ = +0.93, *p* = 0.696) or social resource utilization (Δ = −0.07, *p* = 0.806). EQ-5D utility is summarized descriptively by respondent type in the text and is not tested inferentially because the youth and adult forms are not comparable; the EQ-5D VAS (Table 3) serves as the comparable descriptor.

**Table 4:** Changes before and after the event (paired *t* test, *n* = 15 pairs).

Indicator	*n*	Before M ± SD	After M ± SD	Δ	95% CI of Δ	*t*(14)	*p*	*d*
FES	15	30.5 ± 10.2	31.4 ± 9.2	+0.93	[−4.08, +5.95]	+0.40	0.696	+0.10
EQ-5D VAS	15	72.3 ± 17.6	80.3 ± 14.6	+7.9	[−7.2, +23.0]	+1.13	0.28	+0.29
J-ZBI_8	15	9.1 ± 4.9	13.8 ± 8.1	+4.73	[+0.96, +8.51]	+2.69	0.018*	+0.69
Social resource utilization	15	2.93 ± 0.88	2.87 ± 1.06	−0.07	[−0.64, +0.51]	−0.25	0.806	−0.06

Δ = Post − Pre; 95% CI = 95% confidence interval of the mean change; *d* = Cohen’s *d* (standardized by the SD of the change scores). Estimates (Δ with 95% CI) are emphasized over *p* values given the exploratory, small-sample design. *p < 0.05.

### 3.6 Exploratory analyses

Health-related quality of life is summarized descriptively by respondent type because the two EQ-5D forms are not comparable. For the EQ-5D VAS, the T0–T1 change was concentrated among parent-proxy reports (*n* = 7; Δ = +16.3) and was negligible among sibling self-reports (*n* = 8; Δ = +0.6). The descriptive EQ-5D utility data showed the same pattern (parent-proxy Δ = +0.084; sibling self-report Δ = +0.034). Thus, any apparent post-event improvement in quality of life reflected mainly parents’ perceptions of their children rather than the children’s own ratings, and should be regarded as exploratory.

The following are exploratory analyses intended to generate hypotheses.

#### 3.6.1 Association between Zarit change pattern and RCS service use

Table 5 shows RCS service use for cases in which J-ZBI_8 increased from T0 to T1 (Zarit-increase group; *n* = 9) and cases in which it decreased or remained unchanged (*n* = 6). Whereas 2 cases (22%) in the Zarit-increase group had used the RCS, 5 cases (83%) in the decrease/no-change group had done so, the point estimate suggested a possible association (OR = 0.06, 95% CI [0.004, 0.82]) but was highly unstable given the very small cell counts and is reported as hypothesis-generating only. In addition, two cases newly registered with the RCS after the event; this is reported as a descriptive count, not as evidence of an entry-point function.

**Table 5:** RCS service use by Zarit change group (exploratory analysis, *n* = 15).

Group	RCS used	RCS not used	Total
Zarit-increase group (*n* = 9)	2 (22%)	7 (78%)	9
Decrease/no-change group (*n* = 6)	5 (83%)	1 (17%)	6
Total	7	8	15

“Used” denotes use of at least one of the four RCS services (FEP, peer salon, individual consultation, webinar). Cell counts are small; group percentages are descriptive.

#### 3.6.2 Comparison by care recipient severity

Based on required level of assistance, near-total/total assistance was defined as the severe group (*n* = 10) and none/partial assistance as the mild group (*n* = 9), and indicators were compared (Table 6). At T0, the severe group had significantly higher social resource utilization than the mild group (3.38 ± 0.74 vs. 2.50 ± 0.76, *p* = 0.041) and tended to have lower J-ZBI_8 (7.1 ± 5.5 vs. 11.0 ± 2.9, *p* = 0.058). That is, before the event, levels of social resource utilization and caregiver burden differed by care recipient severity. For change scores (Δ), however, no significant between-group differences were found for any indicator. Social resource utilization in the severe group decreased slightly toward T1 (Δ = −0.25), whereas the mild group increased slightly (Δ = +0.14). As a result, the between-group difference narrowed from 0.88 points at T0 to 0.48 points at T1 and was no longer statistically significant.

**Table 6:** Comparison of indicators by care recipient severity (required level of assistance; exploratory analysis).

Indicator	Severe group M ± SD (*n*)	Mild group M ± SD (*n*)	*p*
Before event (T0)			
FES	34.3 ± 12.1 (8)	27.5 ± 6.4 (8)	0.083
J-ZBI_8	7.1 ± 5.5 (8)	11.0 ± 2.9 (8)	0.058
Social resource utilization	3.38 ± 0.74 (8)	2.50 ± 0.76 (8)	0.041*
After event (T1)			
FES	31.8 ± 11.6 (9)	28.6 ± 7.1 (8)	0.354
J-ZBI_8	12.6 ± 9.3 (9)	16.5 ± 8.9 (8)	0.497
Social resource utilization	3.11 ± 1.05 (9)	2.63 ± 0.92 (8)	0.265
Change score Δ (T1 − T0)			
FES	−0.25 ± 7.82 (8)	+2.29 ± 10.78 (7)	0.816
J-ZBI_8	+6.25 ± 7.91 (8)	+3.00 ± 5.39 (7)	0.485
Social resource utilization	−0.25 ± 0.71 (8)	+0.14 ± 1.35 (7)	0.748

Severe group, near-total/total assistance (*n* = 10); mild group, none/partial assistance (*n* = 9). *p*-values are from exploratory, unadjusted Mann–Whitney U tests (two-sided) and are hypothesis-generating; *p < 0.05.

## 4 Discussion

This pilot study provides preliminary, primarily feasibility-oriented evidence on a hybrid model of sibling support in which an in-person peer event is embedded within a continuously operating online remote care system (RCS). Although no progression criteria were prespecified, we benchmarked the observed indicators *post hoc* against thresholds used in the pilot-trial literature, recognizing that such targets vary across studies and are commonly operationalized with a red/amber/green (“go/amend/stop”) framework (Eldridge et al., 2016; Herbert et al., 2019); for interpretive purposes we adopted recruitment/participation ≥ 80%, retention ≥ 70%, and acceptability ≥ 75%. Participation (91%) and acceptability (all children rated the event enjoyable; 94.7% wished to attend again) met these benchmarks, whereas questionnaire retention fell short (53% at 3 months and 63% at 6 months). Mapped onto such a framework, this profile corresponds to an “amber/amend” decision: the model warrants progression to a definitive trial only if retention procedures are strengthened and progression criteria are prespecified. The model could be delivered and followed over 6 months: recruitment was open, participation was high (20 of 22 applicants), and no adverse events were reported, although questionnaire follow-up declined markedly at 3 and 6 months. The model differs from the predominantly in-person, standalone programs that characterize the existing literature (Al-Hakeem et al., 2025) in coupling episodic in-person connection with continuous, geographically unconstrained remote support, so that the event is one node within an ongoing support relationship rather than an isolated encounter. The present data are insufficient to establish efficacy, but they are consistent with the feasibility of integrating episodic in-person sibling events into a continuous remote family-support infrastructure and identify the measurement and retention challenges that future controlled evaluations must address.

### 4.1 Characteristics of the participants

The 19 cases encompassed diverse disability types among care recipients—autism spectrum disorder/pervasive developmental disorder (*n* = 7), cerebral palsy (*n* = 4), chromosomal abnormalities/rare diseases (*n* = 4), and intellectual disability/developmental delay (*n* = 3). Required level of assistance was roughly evenly split between severe and mild, reflecting the diversity of the population that community-based sibling support should target.

### 4.2 Baseline characteristics and descriptive trends

Baseline scores and their trends over time are summarized in Table 3. Given the small sample, the two non-comparable EQ-5D forms, and high attrition at T2 and T3, values beyond T1 are presented as descriptive trends only and are not interpreted as evidence of maintained change.

The T0 J-ZBI_8 (9.0 ± 4.7) was somewhat lower than the reference values for home caregivers reported by Arai et al. (2003; approximately 11–14 points). However, because there was no control group and most participants (18 of 20) registered with the RCS in connection with the event rather than having received continuous prior support, this difference cannot be attributed to RCS involvement. FES (30.9) corresponded almost exactly to the scale midpoint (30), indicating a moderate level of empowerment. Social resource utilization (2.94 of 4) slightly exceeded the scale midpoint (2.5) and is reported as a baseline descriptor only.

### 4.3 Changes before and after the event

Quality of life was treated as an exploratory secondary outcome. On the EQ-5D VAS—the only quality-of-life metric comparable across forms—the T0–T1 change was small and non-significant (Δ = +7.9, *p* = 0.28, d = 0.29) and was concentrated among parent-proxy reports, with essentially no change in children’s self-reports; the descriptive EQ-5D utility data showed the same pattern. Because the sample was small and uncontrolled, the youth and adult EQ-5D forms are not comparable, and parent-proxy and child self-reports may diverge, these patterns are hypothesis-generating only and cannot be attributed to the event. At 3 and 6 months, high missingness precludes interpretation, and the data are presented as descriptive trends. This caution is consistent with prior sibling-focused work: systematic reviews of sibling-support programs have reported potential psychosocial benefits, including effects on emotional outcomes such as anxiety and mood, although effects remain inconsistent across studies (Hartling et al., 2014; Wolff et al., 2023), against a background of elevated psychosocial risk among siblings (Stoneman, 2005).

J-ZBI_8 also increased immediately after the event (mean increase +4.73, 95% CI [0.96, 8.51]) (Δ = +4.73, *p* = 0.018, d = 0.69). Furthermore, descriptive statistics showed an inverted-U pattern in which the increase continued through 3 months (15.3) and then converged to the T0 level (9.0) by 6 months (8.2) (Figure 2; statistical tests were not performed for T2 and T3 because of the high rates of missing data). One possible interpretation is that emotional expression during the sibling work increased awareness of caregiving-related concerns; however, the present data cannot distinguish this explanation from a genuine increase in perceived burden. A comparable temporal pattern has been reported for a therapeutic recreation camp serving children with chronic illness and their siblings, in which longer-term psychosocial gains were preceded by short-term adverse effects (Kiernan et al., 2004); this parallel is consistent with the possibility that emotionally activating group experiences transiently heighten distress before improvement. Alternatively, an actual increase in burden associated with event participation (e.g., travel and accompaniment) cannot be ruled out.

No significant changes were found in FES (Δ = +0.93, *p* = 0.696) or social resource utilization (Δ = −0.07, *p* = 0.806) before and after the event. As an exploratory secondary outcome, family empowerment would not be expected to shift immediately; as Koren et al. (1992) noted, empowerment is cultivated through a long-term, staged process and is therefore unlikely to change in the short term after a single event. Prior studies of Japanese families raising children with disabilities indicate that family empowerment is associated with awareness and use of social support, problem-solving, and family bonding (Wakimizu et al., 2011, 2016, 2018); accordingly, repeated peer contact combined with continued RCS engagement might, over time, foster these antecedents of empowerment—a hypothesis-generating possibility that warrants longer-term evaluation. Social resource utilization was already at a certain level at T0 (2.94), which may have limited the room for change.

### 4.4 Interpretation of the exploratory analyses

Regarding RCS service use, the point estimate suggested that the Zarit-increase group had engaged with RCS services less often (22%) than the decrease/no-change group (83%) (OR = 0.06, 95% CI [0.004, 0.82]); given the very small cell counts, this estimate is highly unstable. Because RCS use here reflects cumulative engagement after the event (recorded as of the March 2026 log), it postdates the T0–T1 burden change and therefore cannot have caused it; any association more plausibly reflects lower-burden caregivers subsequently engaging more, or confounding, and is hypothesis-generating only. With *n* = 15, this analysis is small in scale, and a definitive interpretation requires future large-scale studies. Given the small sample and the exploratory, statistically unstable nature of this association, it should be regarded as hypothesis-generating only.

In the severity-based analysis, at T0 the severe group had significantly higher social resource utilization (*p* = 0.041) and tended to have lower J-ZBI_8 (*p* = 0.058). Because caregivers of children with more severe disabilities require more specialized support from an earlier stage, social resource utilization may have been promoted even before event participation; the accompanying lower J-ZBI_8 in this group may reflect differences in support access rather than a true difference in caregiver burden. For change scores (Δ), no significant differences were found between the severe and mild groups. For social resource utilization, although the severe group was significantly higher at T0, after the event the severe group decreased slightly while the mild group increased slightly—a convergence pattern. This suggests that the severe group had little room for change because it had been using social resources sufficiently before the event, whereas the mild group, with relatively lower utilization, may have broadened its access to social resources following the event. In other words, the two groups followed different pathways but converged to similar levels after the event. For J-ZBI_8 change scores, no significant difference was found between the groups, and the pattern of change in caregiver burden was similar regardless of severity. With *n* = 9–10, this exploratory analysis is small in scale and is positioned as preliminary, hypothesis-generating findings.

### 4.5 Limitations and future directions

First, the sample was small (*n* = 19; T0–T1 analyses *n* = 15) and follow-up was incomplete at T2 and T3 (47–53% missing), so the study was underpowered for hypothesis testing; outcome analyses are exploratory and intended to inform the design and sample size of future trials. Attrition was non-random: cases completing the 6-month follow-up had higher baseline family empowerment than non-completers (Mann–Whitney *p* = 0.03), so later-wave findings may not generalize to less-empowered families and are presented descriptively. Second, the single-group pre–post design without a control group means observed changes cannot be attributed to the event (regression to the mean, maturation, and seasonal effects cannot be excluded). Moreover, because 18 of the 20 participants registered with the RCS around the time of the event, exposure to the in-person event and subsequent RCS support overlapped temporally; their respective contributions cannot be disentangled, and this applies to all observed outcome patterns. Third, participants were RCS registrants from specific regions of eastern Japan, limiting representativeness. Fourth, the exploratory association between RCS use and burden change rested on very small cell counts and did not control for confounders (e.g., registration period, frequency of use); it is unstable and prone to sparse-data bias. Fifth, outcomes combined parent-proxy (10 cases) and sibling self-reports (9 cases), which capture perceived rather than objective status and may diverge; one record represented a sibling pair sharing a single proxy report. Sixth, health-related quality of life was assessed with two non-interchangeable EQ-5D forms (EQ-5D-Y for adolescent self-reports, adult EQ-5D-5L for parent proxy reports) scored with value sets on different scales; utility values are therefore reported descriptively by respondent type rather than pooled, and the EQ-5D VAS (common to both forms) was used as the comparable descriptor. Seventh, the J-ZBI_8 was developed and validated primarily for caregivers of older adults and was completed by a mix of parents and siblings; its construct validity for young carers is uncertain, and a young-carer–specific measure would be preferable. Eighth, the RCS was developed and is operated by our research group, which also organized the event; this dual role may introduce evaluation bias that independent replication should address. The single-item social resource measure also lacks established psychometric properties. Acceptability was assessed immediately after the event with brief, non-validated satisfaction items collected on-site at a researcher-organized event, and is therefore susceptible to ceiling effects and social desirability or courtesy bias. Implementation fidelity was not assessed with a formal checklist, and per-component participation was not systematically recorded (although delivery of all components and the absence of mid-event withdrawal were confirmed). As a pilot, no formal progression criteria (prespecified thresholds for recruitment, retention, or acceptability) were established *a priori*, and reasons for follow-up non-response at 3 and 6 months were not systematically recorded; future feasibility trials should address both. Future studies should also consider more clinically homogeneous samples or stratification by care recipient condition and severity; where appropriate, prior RCS exposure should be considered in the study design to better distinguish the contribution of the in-person event from that of ongoing RCS support. In light of these considerations, this study is positioned as a feasibility pilot providing preliminary, hypothesis-generating findings to inform future controlled trials.

## 5 Conclusion

This pilot indicates that an in-person sibling peer event embedded within a continuously operating online remote care system can be delivered and followed over 6 months, with open recruitment, high participation, and no reported adverse events, while also exposing substantial follow-up attrition by 3 and 6 months. Exploratory outcome patterns—small, non-significant, mainly parent-proxy changes in quality of life, and a transient post-event rise in caregiver burden that returned toward baseline—are hypothesis-generating only and cannot be attributed to the event in this uncontrolled design. The main contribution is methodological: the study illustrates the feasibility of this hybrid model and the measurement and retention challenges—non-comparable EQ-5D forms, mixed proxy and self-report respondents, and high attrition—that future controlled trials of sibling support should be designed to overcome.

## Data Availability

The raw data supporting the conclusions of this article will be made available by the authors, without undue reservation.
