# Supplementary material for: Feasibility of a remote-care-system-based sibling peer event for young carers: a longitudinal pilot study
Source: Front Psychol. 2026 Sep 30;17:1940533. doi: 10.3389/fpsyg.2026.1940533 (PMC13661563; doi:10.3389/fpsyg.2026.1940533)
Supplement: Supplementary file 1 [file Table_1.docx]

**TREND Statement Checklist**

*Feasibility of a Remote-Care-System-Based Sibling Peer Event for Young Carers: A Longitudinal Pilot Study*

This single-group, non-randomized feasibility pilot is reported in line with the TREND statement (Des Jarlais et al., 2004) for non-randomized evaluations. Pilot- and feasibility-specific elements (e.g., progression considerations) additionally follow the principles of the CONSORT extension for pilot and feasibility trials (Eldridge et al., 2016). Locations refer to the numbered manuscript sections of the submitted version; page and line numbers are available in the manuscript file.

| **No.** | **TREND item (paraphrased)** | **Reported in manuscript** |
| --- | --- | --- |
| 1 | Title/abstract: study design (single-group, non-randomized), target sample, and structured summary of methods and results. | Title; Abstract |
| 2 | Scientific background, rationale, and theoretical basis (family empowerment; hybrid in-person + continuous online support). | 1 Introduction |
| 3 | Participants: eligibility criteria, recruitment method, and settings/locations. | 2.4 Participants; 2.2 Study Setting: The Remote Care System |
| 4 | Interventions: content, mode and timing of delivery, and underlying rationale. | 2.3 Intervention; 2.2 Study Setting: The Remote Care System |
| 5 | Objectives and aims (primary: feasibility; exploratory secondary: outcomes). | 1 Introduction (aims); 2.7 Statistical Analysis |
| 6 | Outcomes: clear definitions, assessment methods, and instrument validity (FES, EQ-5D, J-ZBI_8, social resource use; feasibility indicators). | 2.6 Measures; 2.5 Assessment Method; Table 2 |
| 7 | Sample size: pilot study; no formal power calculation (rationale and implications noted). | 2.7 Statistical Analysis; 4.5 Limitations and Future Directions |
| 8 | Assignment method: single-group design with no randomization (one case per participating sibling). | 2.1 Study Design; 2.4 Participants |
| 9 | Blinding (masking): not applicable to a single-group design; self- vs proxy-report specified. | 2.5 Assessment Method (N/A; respondent type specified) |
| 10 | Unit of analysis (one case per sibling; sibling pair collapsed to one record). | 2.4 Participants; 2.7 Statistical Analysis |
| 11 | Statistical methods, including feasibility benchmarks and exploratory analyses (estimates with 95% CIs). | 2.7 Statistical Analysis |
| 12 | Participant flow (applied, attended, analyzed; attrition by time point). | 3.1 Participant Overview; Figure 2; Table 2 |
| 13 | Recruitment and follow-up timing (event date; T0–T3 schedule). | 2.3 Intervention; 3.1 Participant Overview; Table 2 |
| 14 | Baseline participant and care-recipient characteristics. | 3.1 Participant Overview; Table 1 |
| 15 | Baseline comparability across subgroups (severity strata). | 3.4 Exploratory Analyses; Table 6 |
| 16 | Numbers analyzed at each time point (denominators stated). | 3.2 Descriptive Statistics for Each Indicator; Tables 2–3 |
| 17 | Outcomes and estimation: effect estimates with 95% CIs (feasibility primary; outcomes exploratory). | 3.3 Changes Before and After the Event (T0–T1); Tables 2–4; Figure 1 |
| 18 | Ancillary/exploratory analyses (RCS service use by burden change; severity comparison). | 3.4 Exploratory Analyses; Tables 5–6 |
| 19 | Adverse events / unintended effects. | 3.1 Participant Overview; Table 2 (none reported) |
| 20 | Interpretation of results, consistent with the feasibility (not efficacy) aim; hypothesis-generating outcomes. | 4 Discussion |
| 21 | Generalizability (RCS-registered, single-site, small sample). | 4.5 Limitations and Future Directions |
| 22 | Overall evidence and progression considerations toward a future definitive trial. | 4.5 Limitations and Future Directions; 4.6 Conclusion |

*Note. Item descriptors are paraphrased. The study is non-randomized and single-group; randomization- and blinding-specific items are marked not applicable.*
